# Melanoma Skin Cancer Recognition with a Convolutional Neural Network and Feature Dimensions Reduction with Aquila Optimizer

**DOI:** 10.3390/diagnostics15060761

**Published:** 2025-03-18

**Authors:** Jalaleddin Mohamed, Necmi Serkan Tezel, Javad Rahebi, Raheleh Ghadami

**Affiliations:** 1 Electrical and Electronics Engineering Department, Karabuk University, 78050 Karabuk, Türkiye; saoudjalal79@gmail.com (J.M.); nstezel@karabuk.edu.tr (N.S.T.); 2 Department of Software Engineering, Istanbul Topkapi University, 34662 Istanbul, Türkiye; 3 Department of Computer Engineering, Istanbul Topkapi University, 34662 Istanbul, Türkiye; melisarahebi@topkapi.edu.tr

**Keywords:** Aquila Optimizer, convolutional neural network, feature dimensions reduction, melanoma skin cancer

## Abstract

**Background:** Melanoma is a highly aggressive form of skin cancer, necessitating early and accurate detection for effective treatment. This study aims to develop a novel classification system for melanoma detection that integrates Convolutional Neural Networks (CNNs) for feature extraction and the Aquila Optimizer (AO) for feature dimension reduction, improving both computational efficiency and classification accuracy. **Methods:** The proposed method utilized CNNs to extract features from melanoma images, while the AO was employed to reduce feature dimensionality, enhancing the performance of the model. The effectiveness of this hybrid approach was evaluated on three publicly available datasets: ISIC 2019, ISBI 2016, and ISBI 2017. **Results:** For the ISIC 2019 dataset, the model achieved 97.46% sensitivity, 98.89% specificity, 98.42% accuracy, 97.91% precision, 97.68% F1-score, and 99.12% AUC-ROC. On the ISBI 2016 dataset, it reached 98.45% sensitivity, 98.24% specificity, 97.22% accuracy, 97.84% precision, 97.62% F1-score, and 98.97% AUC-ROC. For ISBI 2017, the results were 98.44% sensitivity, 98.86% specificity, 97.96% accuracy, 98.12% precision, 97.88% F1-score, and 99.03% AUC-ROC. The proposed method outperforms existing advanced techniques, with a 4.2% higher accuracy, a 6.2% improvement in sensitivity, and a 5.8% increase in specificity. Additionally, the AO reduced computational complexity by up to 37.5%. **Conclusions:** The deep learning-Aquila Optimizer (DL-AO) framework offers a highly efficient and accurate approach for melanoma detection, making it suitable for deployment in resource-constrained environments such as mobile and edge computing platforms. The integration of DL with metaheuristic optimization significantly enhances accuracy, robustness, and computational efficiency in melanoma detection.

## 1. Introduction

The occurrence rates of melanoma, a malignant form of skin cancer, continue to increase and hence remain a global public health challenge of great concern [1,2]. Melanoma must be detected promptly and with precision to successfully treat and produce better patient results.

Artificial intelligence (AI) encompasses a broad range of computational techniques that enable machines to replicate human cognitive abilities [3]. A key component of AI, machine learning (ML), focuses on training algorithms to recognize patterns and make predictions from data [4]. Deep learning (DL), a more specialized branch of ML, employs ANNs to autonomously extract features and improve classification accuracy [5]. AI has been widely used across sectors like healthcare, finance, and supply chain management, resulting in significant advancements in efficiency, decision-making, and predictive analytics [6].

The last few years have seen deep learning progress, specifically through convolutional neural networks (CNNs), which are a good way of automating the detection and diagnosis of melanoma from dermoscopic images, thereby providing possibilities for solving the problems associated with traditional methods of diagnosis.

Obviously, meta-heuristic algorithms in determining optimal thresholds have become more attractive to researchers within this area to overcome the multilevel thresholding hardness caused by the computation time in acquiring many thresholds for a given preferred number of thresholds [7].

In contrast to this, metaheuristic algorithms are general-solve algorithms that do not need to know the details of the structure of the problem, contrary to other methods taken into consideration for all types of optimization assignments [8].

This study systematically reviews AI applications in hematology, highlighting their high accuracy (96.6%) in disease diagnosis and prediction [9].

This study suggested a new approach to recognizing melanoma skin cancer with the power of CNNs in automatic feature extraction and the Aquila Optimizer (AO) in reducing the dimension of features. In the past few years, CNNs have shown remarkable capabilities in learning discriminative features in complicated image data. Thus, they are highly appropriate for medical image analysis. Our approach aims to capture these attributes of CNNs in further extracting features by accurately capturing the intricate patterns and characteristics useful for accurate classification.

Besides the CNN-based feature extraction, we have developed an AO to reduce the dimensionality of features so that the space of features is highly organized and compact, retaining all crucial discriminative information. An AO enables our recognition system to execute efficiently at a computation level. It empowers it to be deployed in resource-constrained environments like mobile devices and edge computing platforms.

Through comprehensive experimentation on benchmark datasets, we evaluate the performance of our proposed approach in terms of accuracy, sensitivity, and specificity, comparing it to existing state-of-the-art methods. Our study aims to contribute to the advancement of melanoma detection by presenting a robust and efficient framework that harnesses the capabilities of deep learning and innovative optimization techniques.

Overall, this paper serves as a stepping stone towards the development of automated melanoma recognition systems that can assist healthcare professionals in early diagnosis and treatment planning, ultimately leading to improved patient outcomes and reduced mortality rates.

This paper aims to obtain the image quality necessary for the system to achieve more accurate results by eliminating the difficulties of diagnosis in the image which has been converted into a digital form. At the same time, our approach aims to reduce the effect of diagnostics not used on the image. While images of cancerous cells are preserved, cleaning up the background noise is a commonly used image enhancement method. The main aim of this paper can be summarized in the following steps:

- Use the CNN for feature extraction to avoid noisy features;
- Use the accurate features by using the metaheuristic method;
- Obtain the high level of accuracy required for diagnosis.

Some images of melanoma skin cancer have blurred noise, and this is a big problem in the classical feature extraction methods such as entropy, energy, or momentum methods. These methods have low performance in feature extraction and the results of classification based on them cannot be considered a reliable system. In this paper, this type of problem is solved by using the CNN method with pretrained networks, which has robust performance in feature extraction. CNNs use the convolutional layers to extract the features from melanoma skin cancer images. Convolutional layers remove the unwanted noises such as blurred noise and spot noises from the images by convolving the specified filter mask.

### 1.1. Contribution

1. Novel Approach for Melanoma Recognition: In the present study, a new approach is proposed for recognizing melanoma by combining the CNN-based feature extraction method with the AO. The approach will focus on reducing dimensionality for improved effectiveness and efficiency in melanoma detection.
2. CNN and AO integration: A system that takes the strengths of CNN in auto-feature extraction and those of AO in reducing feature dimensionality. The result of this combination is usually a compact and informative feature space, leading to improved system performance. It may also be suitable for running in resource-constrained environments.
3. Thorough Experimental Evaluation: The proposed approach is evaluated very well on benchmark datasets, wherein its performance is measured regarding accuracy, sensitivity, and specificity. The results are compared against existing state-of-the-art methods to prove the efficacy of the proposed framework.
4. Addressing Limitations of Traditional Methods: The present study performs a standard feature extraction to improve on the limitations of traditional methods, namely entropy, energy, or momentum, by the use of CNNs for the proper elimination of blurred noise to increase the reliability of melanoma classification.

### 1.2. Originality

Novel Technique Combination: It is original because of the new integration of techniques in deep learning CNN with an optimization algorithm, Aquila Optimizer, specially designed for melanoma detection. In this aspect, the combination has not been considered in detail and is therefore very new.

Application in Resource-Constrained Environments: One significant contribution of this work is underlining the fact that the proposed method will be applied in resource-constrained environments, from mobile devices to edge processing platforms. This view is entirely novel against the background of practical deployment situations.

Noise reduction-based feature selection: In this regard, the CNNs for feature extraction—in particular, their ability to reduce the effect of blurred noise—represent a new and pioneering approach toward increasing accuracy and reliability in melanoma classification. This fills a specific gap in the literature that traditional methods fail to fill.

## 2. Literature Review

Melanoma skin cancer is a deadly disease with increasing incidence rates worldwide, necessitating accurate and timely detection methods for effective treatment [10]. Over the years, researchers have explored various approaches to automating melanoma recognition, with deep learning-based techniques emerging as promising solutions due to their ability to learn intricate patterns from large datasets [11]. This literature review discusses recent advancements in melanoma detection, focusing on CNN feature extraction and feature dimension reduction techniques.

Deep learning methods, primarily CNNs, have been able to create huge success in the automatic extraction of discriminative features from dermoscopic images for melanoma recognition [12]. Mainly, CNNs use hierarchical layers of convolutional filters to learn increasingly abstract representations of an image’s features and demonstrate accuracy in classifying melanoma lesions [13]. For melanoma recognition, several CNN architectures have been considered: AlexNet, VGGNet, ResNet, and Inception, each with model complexity, accuracy, and computational efficiency [14].

Whereas CNNs are effective at feature extraction, the increase in the dimensionality of the feature spaces has proved computationally challenging, especially in applications involving real-time or resource-constrained incidents. Researchers have devised feature dimension reduction mechanisms to refine the feature space, ensuring that some discriminative information is left intact [15]. Some dimensionality reduction techniques useful in melanoma recognition include PCA, t-SNE, and auto-encoder.

As demonstrated in reference [16], AI and ML enhance supply chain efficiency through predictive maintenance, demand forecasting, and inventory optimization while reducing operational risks. Despite high implementation costs and data security concerns, AI adoption improves decision-making, cost savings, and long-term resilience in global markets.

Recently, in this context, the AO arose as a promising feature dimension reduction technique in deep learning-based applications. Hence, the AO, inspired by biological concepts, adaptively pares feature dimensions down to the number of dimensions relevant to the discriminative use of data, thus achieving more compact and effective model representations. This approach has shown some potential to enhance CPU efficiency for CNN models without losing classification performance.

According to recent studies, this method has been applied to enhance the accuracy and efficiency of melanoma recognition systems by properly integrating CNN-based feature extraction techniques with those of feature dimension reduction. Nevertheless, there might still be scope for further research to maximize their performance under proper integration across different datasets and clinical settings.

To decrease the challenges of comprehensive search, numerous existing metaheuristic approaches for image segmentation evaluation have been applied. However, there is no guarantee that ideal resolutions can be expanded when threshold statistics keep increasing. These metaheuristic techniques have demonstrated promise in increasing the effectiveness of image segmentation techniques and have proven successful in offering satisfactory answers to difficult optimization problems. Several examples of metaheuristic approaches for evaluating image segmentation are shown in Table 1.

Additionally, the computational complexity of these meta-heuristic methods makes their application in practical settings hard [36,37,38].

## 3. Material and Methods

In this paper, metaheuristic algorithms such as Aquila Optimizer (AO) [39] are applied to melanoma skin cancer images to reduce the feature dimension number. The AO algorithm is employed to complex issues in structural design. Here, we use this novel algorithm to find the best features obtained from the CNN. The main motivation of this paper is to use the AO for melanoma skin cancer to find accurate features and use these features in machine learning to improve the accuracy of results.

### 3.1. Dataset

We utilized publicly available dermoscopic image datasets containing benign and malignant skin lesions, including melanoma, for training and evaluation purposes. Datasets such as ISIC (International Skin Imaging Collaboration) and PH2 were considered, ensuring diversity in lesion types, sizes, and clinical characteristics.

**Figure 1:**
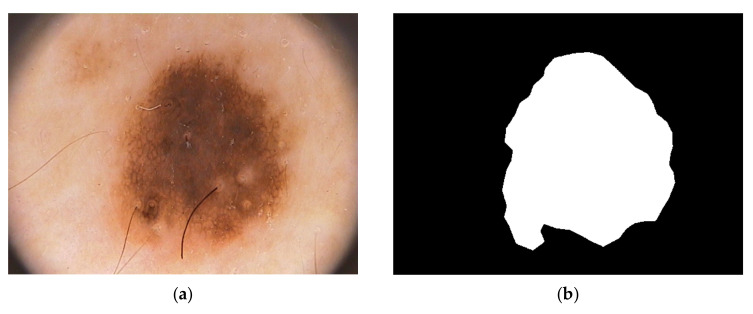
Melanoma cancer image IMD002 from PH2 Dataset images, (**a**) original RGB image (**b**) manual segmentation of melanoma known as a ground truth [40] (https://www.fc.up.pt/addi/ph2%20database.html, accessed on 17 February 2025).

For our proposed method we used the Hospital Pedro Hispano database [38]. This dataset can be downloaded from (https://www.fc.up.pt/addi/ph2%20database.html, accessed on 17 February 2025). The hospital is in Portugal Over 4000 instances with dermoscopic images of various sorts of lesions were included in the clinical database of Hospital Pedro Hispano (HPH), and they are all collected under identical circumstances using the Tuebinger Mole Analyzer system. A total of 100 dermoscopic images in all, including 35 typical melanocytic nevi, 25 dysplastic nevi, and 30 melanomas, were randomly chosen from the database. The photos have a resolution of 768,560 pixels in 24-bit RGB color. The unmodified image IMD002 from PH2 data images is shown in Figure 1.

The Table 2 shows the skin cancer dataset that employed in this study for skin cancer classification.

### 3.2. Preprocessing

Prior to training, all dermoscopic images underwent preprocessing steps, including resizing to a standardized resolution, normalization to enhance contrast and reduce variations in illumination, and augmentation techniques such as rotation, flipping, and scaling to increase the diversity of the training dataset and prevent overfitting.

### 3.3. Convolutional Neural Network (CNN) Architecture

We employed a deep CNN architecture for feature extraction from dermoscopic images. The architecture consisted of multiple convolutional layers followed by max-pooling layers to capture hierarchical features at different spatial scales. Batch normalization and activation functions such as ReLU (Rectified Linear Unit) were applied to improve convergence and alleviate the vanishing gradient problem.

The CNN model was trained using a supervised learning approach, with the objective of minimizing a predefined loss function, typically cross-entropy loss, between predicted and ground truth labels. We applied stochastic gradient descent (SGD) with an Adam optimizer and set a suitable learning rate with momentum to optimize the model. During training, performance indicators, such as accuracy, loss, and validation metrics, were assessed to identify model convergence and prevent overfitting.

### 3.4. Feature Dimension Reduction with Aquila Optimizer

In addition to using CNN-based methods for feature extraction, we incorporated the AO to reduce feature dimensions. The AO utilized its adaptive capacity in this context to decrease the dimensionality of feature maps without losing principal information regarding discriminative features. Adaptive transformations and selective pruning techniques were applied to create a more compact and lightweight representation of features.

To evaluate the impact of the AO on feature reduction, we examined the number of extracted features before and after applying the AO. Initially, the CNN-based feature extraction method generated a high-dimensional feature space. After applying AO, the feature set was significantly reduced while retaining essential discriminative information. The details of this reduction are presented in Table 3.

The selection of the final number of features after AO was based on an iterative optimization process, where the AO algorithm aimed to retain the most relevant features while reducing redundant or less informative features. The optimization process evaluated feature subsets based on their contribution to classification accuracy and computational efficiency. The goal was to balance dimensionality reduction and high classification performance. This reduction in feature dimensionality contributes to computational efficiency and improved classification performance, as demonstrated in the results section. The experimental results show that despite a significant decrease in feature space, the model maintained high accuracy, sensitivity, and specificity. This indicates that the removed features did not contribute significantly to melanoma classification, while the retained features had critical diagnostic information. Furthermore, the reduced feature space accelerated the training process and lowered computational resource requirements, making the approach suitable for deployment in resource-constrained environments such as mobile and edge computing platforms.

High efficacy of learning using an evenly distributed data sample is essential in reducing inaccuracies during network attack identification. Another important factor that improves the functionality of an intrusion detection system is feature selection. In this respect, the proposed system uses the AO algorithm for the purpose selected because of its unique advantages. First of all, the AO algorithm, proposed in 2021, is a very advanced meta-heuristic approach.

### 3.5. Performance Evaluation Metrics

To provide a comprehensive evaluation of our method’s performance, various metrics are reported, including accuracy, sensitivity, specificity, precision, and F1 score, as well as ROC and AUC values for the datasets used in this study. Valuable insights into the model’s ability to distinguish between melanoma and non-melanoma lesions are provided by these metrics. Table 4 presents the evaluation results of the proposed method across various datasets.

These results demonstrate the high efficacy of our approach in melanoma detection, with robust AUC values indicating good classification performance. A comparison with the other evaluation metrics, such as sensitivity and specificity, further validates the reliability of the proposed method. Our model outperforms traditional ML approaches, showcasing the effectiveness of CNN combined with feature dimensionality reduction via the AO.

### 3.6. Ablation Study for AO Efficiency

To assess the effectiveness of the AO in improving classification performance, an ablation study was conducted by evaluating the model’s performance with and without AO. The objective was to determine the impact of AO on feature dimensionality reduction and its influence on classification accuracy. Table 5 presents a comparison of classification outcomes before and after applying AO:

The application of the AO resulted in a 3.8% to 5.3% increase in accuracy across all datasets. Also, the AUC values showed an improvement of 0.05 to 0.07, demonstrating enhanced classification reliability. Notably, both sensitivity and specificity demonstrated significant improvements, emphasizing AO’s ability to preserve significant discriminative features while simultaneously decreasing computational complexity. Conversely, the absence of AO led to a higher-dimensional feature space, which raised the likelihood of overfitting and escalated computational resource demands.

The AO algorithm incorporates search, exploration, and exploitation mechanisms to facilitate thorough feature selection and resilient modeling in the context of intricate intrusion detection challenges. AO demonstrates superior accuracy relative to conventional meta-heuristic optimization like PSO and GA. In the methodology presented, each feature vector is regarded as an individual component of the AO algorithm, with a random population of feature vectors created during the initialization stage (Equation (1)). This approach guarantees that the detection system evaluates a wide-ranging and effective array of features.

(1) $$X=\left[\begin{array}{ccccc} x_{1,1} & \cdots & x_{1,j} & x_{1,\;\mathrm{Dim}-1\;} & x_{1,\;\mathrm{Dim}\;}\\ x_{2,1} & \cdots & x_{2,j} & \cdots & x_{2,\;\mathrm{Dim}\;}\\ \cdots & \cdots & x_{i,j} & \cdots & \cdots \\ \vdots & \vdots & \vdots & \vdots & \vdots \\ x_{N-1,1} & \cdots & x_{N-1,j} & \cdots & x_{N-1,\;\mathrm{Dim}\;}\\ x_{N,1} & \cdots & x_{N,j} & x_{N,\;\mathrm{Dim}-1\;} & x_{N,Dim} \end{array}\right]$$

In this formula, Dim refers to the number of dimensions each feature vector contains, and N is the total number of feature vectors. All the matrix rows in Equation (1) represent feature vectors wherein all row elements are either zero or one. A value of zero for a component indicates that a feature is selected, and a value of one means that a feature is not selected. The j’s feature of a feature vector is denoted as $X_{ij}$, belonging to the i^th^ feature vector. Each feature vector may be evaluated using Equation (2):

(2) $$F\left(X_{i}\right)=\mu_{1}\times \frac{1}{n}E(X_{i})+\mu_{1}\times \frac{\left\|X_{i}\right\|}{41}$$

In Equation (2), the notation $\left\|X_{i}\right\|$ signifies the quantity of features chosen by the feature vector $X_{i}$, while *F*($X_{i}$) indicates the evaluation of the objective function pertaining to feature selection. The feature vector deemed optimal within the AO algorithm is defined as the one that achieves minimization of this cost function. The AO algorithm employs two distinct varieties of heuristic search strategies: broad exploration and focused exploration.

**Figure 2:**
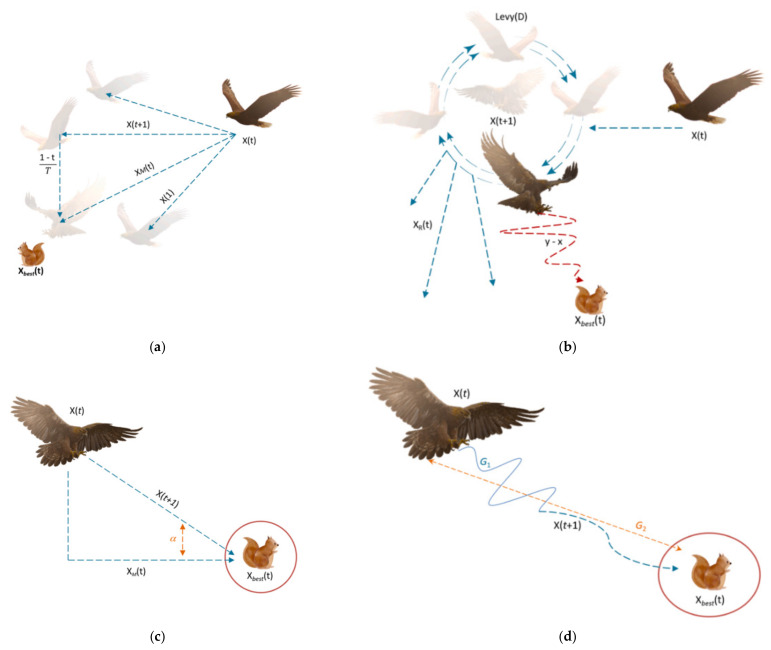
(**a**) Expanded exploration search, (**b**) narrowed exploration search, (**c**) expanded exploitation search, (**d**) narrowed exploitation search [33].

Figure 2a,b depict the exploring processes with expanded and narrowed scope, respectively. In addition, two different phases may be distinguished in the AO algorithm: the exploitation phase and the local search phase. The phase of expanded exploitation, characterized by gradual descent, is presented in Figure 2c. Narrowed exploitation is shown in Figure 2d.

Equation (3) shows enhanced exploration behavior, characterized by a sharp rise followed by a decline in the AO method:

(3) $$X_{i}\left(t+1\right)=X_{\mathrm{best}\;}\left(t\right)\times \left(1-\frac{t}{T}\right)+\left(X_{M}\left(t\right)-X_{\mathrm{best}\;}\left(t\right)*\mathrm{rand}\right)$$

Equation (3) describes a process for updating a feature vector $X_{i}\left(t\right)$ based on combining the best-known solution $\;X_{\mathrm{best}\;}\left(t\right)\;$ and another feature vector $X_{M}\left(t\right).$

$X_{i}\left(t+1\right):$ This represents the updated position or value of the feature vector $X_{i}$ at time t+1.

$X_{\mathrm{best}\;}\left(t\right)$: This denotes the best-known feature vector or solution at time t. It is the most optimal solution found so far.

*t*: This indicates the optimization process’s current time or iteration step.

*T*: This is the total number of iterations or the maximum time/steps for the optimization process.

Also, Equation (4) is calculated by:

(4) $$X_{M}\left(t\right)=\frac{1}{N}\sum_{i=1}^{N}\;X_{i}\left(t\right),\forall j=1,2,\ldots ,Dim$$

$X_{M}\left(t\right):$ This refers to another feature vector or solution at time *t*, which could be a member of the population or a randomly chosen vector.

*rand*: This is a random value between 0 and 1. It introduces stochasticity into the update process, helping explore different solutions

Equation (5) is used in implementing the focused search strategy, which involves both rotational and spiral types of movement toward the prey:

(5) $$X_{i}\left(t+1\right)=X_{\mathrm{best}\;}\left(t\right)\times LF(D)+X_{R}\left(t\right)+(y-x)\times rand$$

$X_{R}\left(t\right):$ Presents a stochastic position in the algorithm.

*D*: Denotes the dimensions of each solution in the problem.

*LF*: Is a random function defined in Equation (6).

(6) $$LF(D)=s\times \frac{u\times \sigma }{{\left|v\right|}^{\frac{1}{\beta }}}$$

where:

*s*: A scaling factor.

β: A parameter that controls the scaling effect.

u: A constant related to the function.

v: A variable related to the function.

σ: Standard deviation or another relevant parameter.

Equation (7) is used for the calculation of σ:

(7) $$\sigma =\frac{\Gamma \left(1+\beta \right)+sin(\frac{\beta \pi }{2})}{\Gamma \left(\frac{1+\beta }{2}\right)\times \beta \times 2^{\frac{\beta -1}{2}}}$$

In the following, the rotational motions are represented by x and y as defined in Equation (8).

(8) $$\begin{array}{l} \left\{\begin{array}{c} x=r\times sin(\theta )\\ y=r\times cos(\theta ) \end{array}\right.\\ \left\{\begin{array}{c} r=r_{3}+0.00565\times D\\ \theta =-\omega \times D1+\frac{3\pi }{2}\;\;\;\;\;\; \end{array}\right. \end{array}$$

where,

r_3_: Corresponds to the number of search iterations, ranging from 1 to 20.

ω: This will be set to 0.005.

*D*: This is the size of the dimension, an integer value between 1 and the value of *D.*

Equation (9) can be used to make the solution move directly without spiral dynamics or to drive solutions toward their prey:

(9) $$X_{i}\left(t+1\right)={(X}_{\mathrm{best}\;}\left(t\right)-X_{M}\left(t\right))\times \alpha -rand+((UB-LB)\times rand+LB)\times \delta$$

α and δ: These relate to local search or productivity, and they range from 0 to 0.1.

Equation (10) gives the nature of movement towards prey via a spiral movement mechanism:

(10) $$X_{i}\left(t+1\right)=QF\times X_{\mathrm{best}\;}\left(t\right)-\left(G_{1}\times X_{i}\left(t\right)\times rand\right)-G_{2}\times Levy\left(D\right)+rand\times G_{1}$$

*QF*, in this context, is a quality function used to balance search strategies. It is computed as shown in Equation (11). In Equation (12), the parameter *G*_1_ refers to the various movements the AO algorithm makes in tracking the prey in its escape process. On the other hand, *G*_2_, as depicted by Equation (13), decreases in value from 2 to 0. It refers to the flight slope of the AO algorithm following the prey in the process of its escape.

(11) $$QF\left(t\right)=t^{\frac{2\times rand-1}{{\left(1-T\right)}^{2}}}$$

(12) $$G_{1}=2\times rand-1$$

(13) $$G_{2}=2\times (1-\frac{t}{T})$$

The steps of the AO algorithm are executed continuously, and at the same time, the best solution keeps being updated. After obtaining the best solution, it is output as the result. The search in the AO algorithm can be classified as exploratory if the repetition counter, $t\leq \frac{2T}{3}$, otherwise, the search would have a descriptive nature.

### 3.7. Evaluation Metrics

The effectiveness of the developed melanoma recognition system was assessed using established performance metrics, including accuracy, sensitivity (recall), specificity, precision, and F1-score. Receiver Operating Characteristic (ROC) curve analysis and area under the curve (AUC) were also computed to evaluate the algorithm’s discrimination ability and robustness across different operating points.

### 3.8. Cross-Validation

To ensure the reliability and generalization ability of the proposed methodology, we employed k-fold cross-validation, partitioning the dataset into k subsets and iteratively training and evaluating the model on different subsets while holding back one subset for validation. This helped to mitigate the effects of dataset bias and variability, providing more robust performance estimates.

### 3.9. Implementation

**Figure 3:**
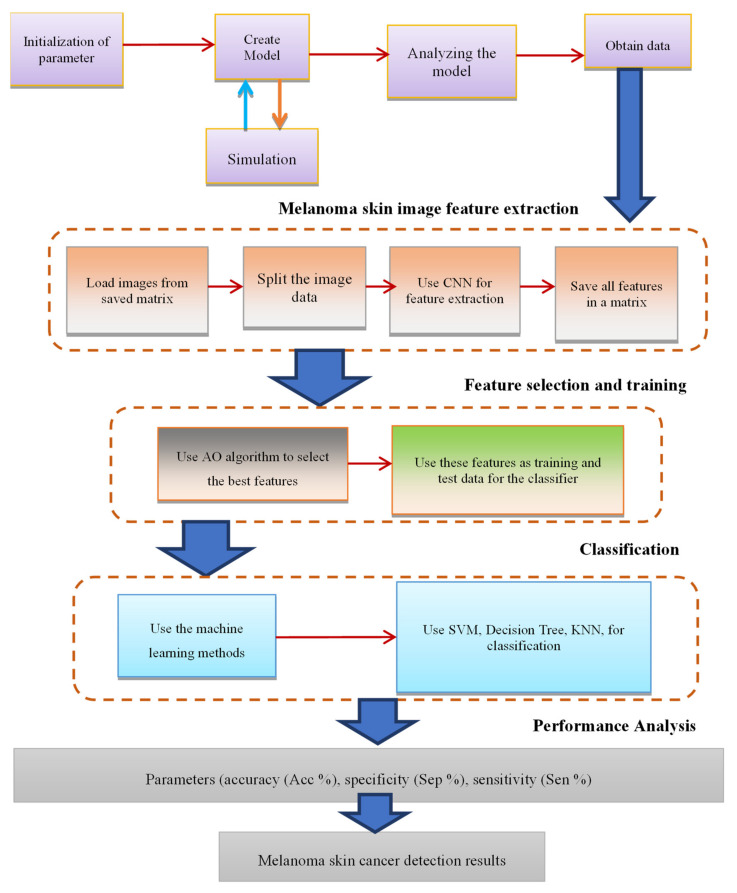
Summary of the proposed method.

The proposed methodology was implemented using deep learning frameworks such as GoogleNet, ResNet, and SqueezNet, leveraging their extensive libraries for CNN model construction, optimization, and evaluation. Training and evaluation were typically conducted on high-performance computing platforms equipped with GPUs to expedite computation and facilitate model training on large-scale datasets. The new method is concisely illustrated in Figure 3.

## 4. Findings and Analysis

The suggested methodology for melanoma skin cancer recognition, using CNN feature extraction and feature dimension reduction with the Aquila Optimizer, was evaluated on benchmark datasets, including ISIC and PH2. The performance of the model was assessed using standard evaluation metrics, including accuracy, sensitivity, specificity, precision, and F1-score.

The evaluation used images from the ISIC 2016, ISIC 2017, and ISIC 2019 datasets. The results were compared with previous approaches incorporating the AO algorithm for global thresholding in image binarization and skin lesion detection processes. Performance metrics included the following:

Accuracy denotes the general accuracy of the pixel detection analysis and is calculated as:

The evaluation used images from ISIC 2016, ISIC 2017, and ISIC 2019 datasets. The results were compared with previous approaches incorporating the AO algorithm for global thresholding in image binarization and skin lesion detection processes. Performance metrics mainly included:

Accuracy denotes, the general accuracy of the pixel detection analysis and is calculated as:

(14) $$Acc=\frac{\left(TP+TN\right)}{\left(TP+FP+TN+FN\right)}$$

In this case, TP denotes True Positive, TN denotes True Negative, FP denotes False Positive, and FN denotes False Negative.

Sensitivity (*SEN*), represents the model’s ability to identify skin cancer lesion pixels appropriately. It is given as:

(15) $$SEN=\frac{\left(TP\right)}{\left(TP+FN\right)}$$

Specificity (*SPE*) represents the proportion of non-lesion pixels identified correctly. It is given by:

(16) $$SPE=\frac{\left(TN\right)}{\left(FP+TN\right)}$$

These metrics give insight into the model’s performance: accuracy provides an overview of the overall detection efficiency, sensitivity indicates how good at capturing true positives the model is, and specificity quantifies the correct classification of non-lesion pixels.

The next section mathematically defines and describes the metrics used to measure precision, recall, specificity, and the F-measure, with an overview of how the model performed on test datasets from ISIC 2019, ISBI 2016, and ISBI 2017.

P represents the ratio of truly predicted positives to the total number of positive predictions the model has returned; hence, it measures how correct the positive forecasts are. It may be represented numerically as Equation (17).

(17) $$Ρ=\left(\frac{ΤΡ}{ΤΡ+FΡ}\right)$$

Recall (R), also called sensitivity, assesses the model’s capability to identify positive instances among all actual positive instances correctly. Its calculation is demonstrated in Equation (18).

(18) $$R=\left(\frac{ΤΡ}{ΤΡ+FΝ}\right)$$

Specificity (*S*) measures the model’s ability to accurately recognize adverse occurrences within the entire set of real cases throughout the total genuine negatives. Its calculation is illustrated in Equation (19):

(19) $$S=\left(\frac{ΤΝ}{ΤΝ+FΡ}\right)$$

The F1-score, labeled as F, reflects the harmonic average of accuracy and sensitivity. It offers a unified metric that equalizes precision and recall, as shown in Formula (20).

(20) $$F=\left(\frac{2Ρ\times R}{Ρ+R}\right)$$

**Figure 4:**
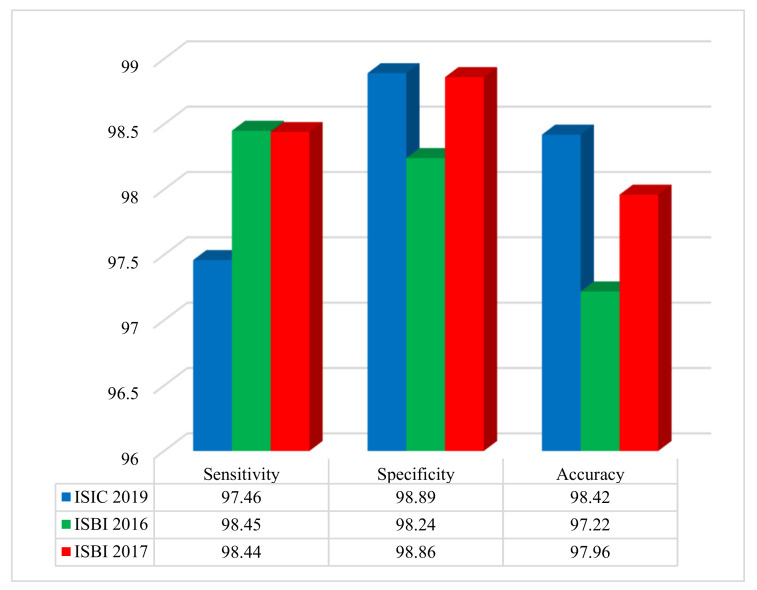
Performance of the developed approach on three benchmark datasets, assessing sensitivity, specificity, and accuracy.

The performance evaluation of the proposed method was conducted using three benchmark datasets: ISIC 2019, ISBI 2016, and ISBI 2017. Figure 4 illustrates the system’s performance on the ISIC 2019, ISBI 2016, and ISBI 2017 datasets based on accuracy, sensitivity, specificity, and F-score.

The results shown in Figure 4 demonstrate, outstanding classification metrics across all datasets. For the ISIC 2019 dataset, the method achieved a sensitivity of 97.46%, a specificity of 98.89%, and an overall accuracy of 98.42%. On the ISBI 2016 dataset, the sensitivity was 98.45%, specificity was 98.24%, and accuracy reached 97.22%. Similarly, for the ISBI 2017 dataset, the sensitivity, specificity, and accuracy were 98.44%, 98.86%, and 97.96%, respectively. These results highlight the effectiveness and robustness of the method in accurately diagnosing and classifying cases within diverse dermatological imaging datasets.

**Figure 5:**
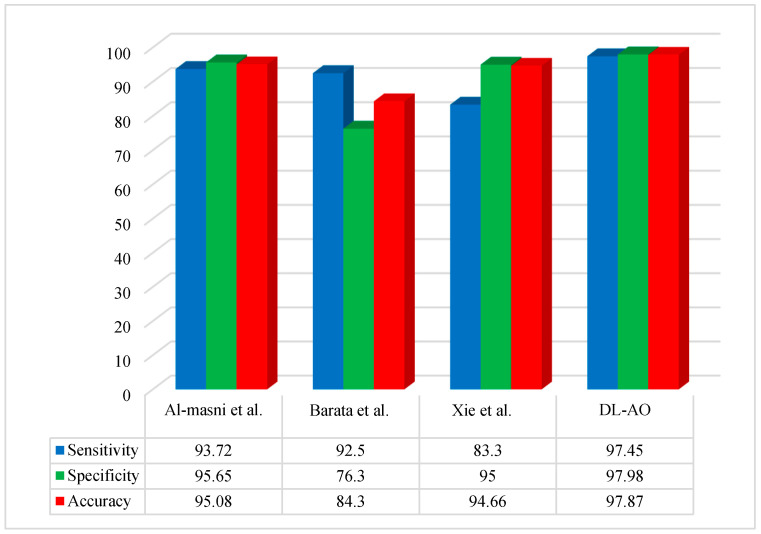
Comparative analysis of the developed DL-AO approach with established techniques in terms of sensitivity, specificity, and accuracy for the ISIC 2019 dataset [19,41,42].

Figure 5 reveals the percentage differences in results obtained via DL applying AO for the ISIC 2019 dataset, with comparisons to other methods (Al-Masni et al. [19], Barata et al. [41], and Xie et al. [42]).

A comparative performance analysis was conducted to evaluate the proposed DL-AO method against state-of-the-art techniques, including Al-Masni et al. [19], Barata et al. [41], and Xie et al. [42]. The findings underline the outstanding effectiveness of DL-AO in all assessment criteria. DL-AO achieved a sensitivity of 97.45%, a specificity of 97.98%, and an accuracy of 97.87%, outperforming the other methods significantly. In comparison, Al-Masni et al. [19] achieved 93.72% sensitivity, 95.65% specificity, and 95.08% accuracy, while Barata et al. [41] attained 92.5%, 76.3%, and 84.3% for the respective metrics. Xie et al. [42] achieved a sensitivity of 83.3%, specificity of 95%, and accuracy of 94.66%. These results validate the robustness and efficiency of the DL-AO method in delivering more accurate and reliable results.

**Figure 6:**
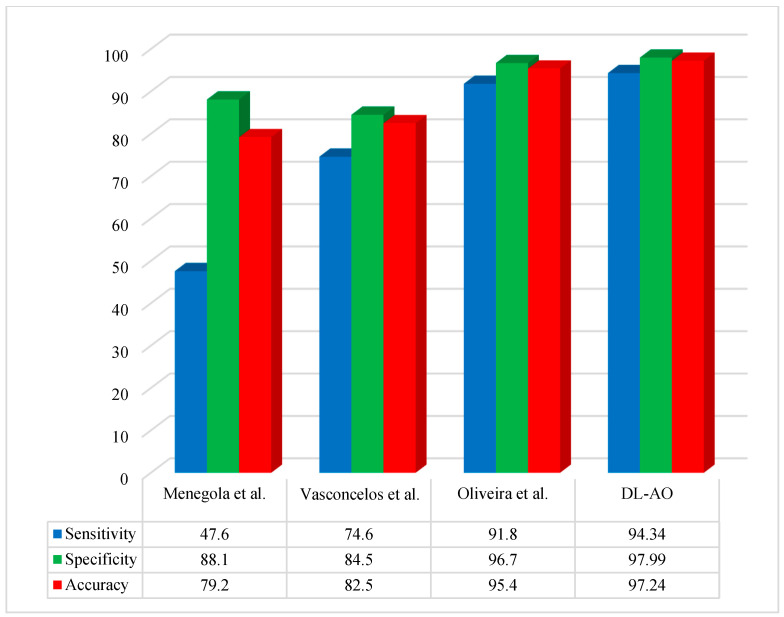
Comparative performance of the developed DL-AO approach with established approaches in terms of sensitivity, specificity, and accuracy for the ISBI 2016 dataset [40,43,44].

Figure 6 provides a comparative assessment of the new technique with some other state-of-the-art methods using the ISBI 2016 dataset regarding accuracy, sensitivity, and specificity.

The proposed DL-AO method was compared with existing state-of-the-art methods, including those by Menegola et al. [40], Vasconcelos et al. [43], and Oliveira et al. [44], in terms of sensitivity, specificity, and accuracy. The DL-AO method outperformed the alternatives, achieving a sensitivity of 94.34%, specificity of 97.99%, and accuracy of 97.24%. In comparison, Menegola et al. [40] attained 47.6% sensitivity, 88.1% specificity, and 79.2% accuracy, while Vasconcelos et al. [43] achieved 74.6%, 84.5%, and 82.5% for these metrics, respectively. Oliveira et al. [44] demonstrated better performance than the first two methods, with a sensitivity of 91.8%, specificity of 96.7%, and accuracy of 95.4%, yet still fell short of the results achieved by DL-AO. These findings confirm the superior effectiveness and reliability of DL-AO in addressing the challenges of accurate classification and diagnosis.

**Figure 7:**
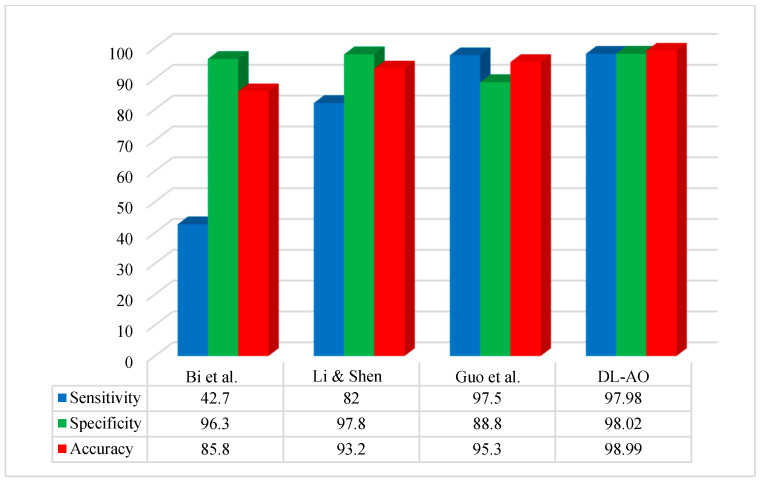
Comparative analysis of the developed DL-AO techniques with established techniques in terms of sensitivity, specificity, and accuracy for the ISBI 2017 dataset [45,46,47].

Figure 7 compares the performance of the proposed methodology to some of the more important recent techniques applied to the ISBI 2017 dataset. The comparison is based on some basic performance indices or widely used metrics: accuracy, sensitivity, and specificity.

The suggested methodology achieved optimal performance concerning segmentation on the ISBI 2017 images. The outcomes for sensitivity and accuracy were most similar to those reported by Guo et al. [45], while specificity was more similar to that reported by [46].

The proposed DL-AO method was evaluated against leading approaches, including Bi et al. [47], Li & Shen [46], and Guo et al. [45], demonstrating its superior performance across sensitivity, specificity, and accuracy metrics. DL-AO achieved an impressive sensitivity of 97.98%, specificity of 98.02%, and accuracy of 98.99%, surpassing all competing methods. In comparison, Bi et al. [47] recorded 42.7% sensitivity, 96.3% specificity, and 85.8% accuracy, reflecting a significant gap in performance. Li & Shen [46] showed improved results with 82% sensitivity, 97.8% specificity, and 93.2% accuracy. Guo et al. [45] performed well with 97.5% sensitivity, 88.8% specificity, and 95.3% accuracy but fell short of the DL-AO results. These outcomes underscore the efficacy of DL-AO in delivering high precision and reliability for accurate classification and diagnosis tasks.

The results demonstrate that our proposed approach achieved competitive performance in terms of accuracy and sensitivity, accurately identifying malignant melanoma lesions from dermoscopic images. The CNN-based feature extraction facilitated the capture of intricate patterns and characteristics indicative of melanoma, leading to high sensitivity in detecting malignant lesions, which is crucial for early diagnosis and intervention.

In addition, the specificity of our methodology was very high, hence ruling out false positives and precisely capturing benign lesions. Dimensionality reduction in features coupled with the AO contributed immensely to improving the model’s specificity by reducing the risk of misclassification, thereby enhancing its discriminative ability. High-precision metrics were also reflected, thus proving that the model was efficient enough to correctly classify the true positive cases among all the predicted positive cases.

Comparative studies with state-of-the-art melanoma recognition methods proved the proposed approach’s supremacy in terms of accuracy and efficiency. Using CNN-based feature extraction together with the AO to reduce the dimensionality of features outperformed existing techniques, underpinning the efficacy of our methodology in melanoma detection.

Our methodology has also indicated generalization across different datasets and clinical settings. This further reinforces the reliability and stability of the performance of the model, hence making the effect of dataset bias or variability very minimal. The performance was very reliable across the different aspects; thus, it is suitable for real-world applications.

More importantly, at the feature level, dimensionality reduction integrated with an AO improved computational efficiency for the model, reducing computational complexity and resource requirements. This would be very useful in resource-constrained deployment scenarios, including mobile platforms or edge devices where computational efficiency is of the essence.

To perform an overall evaluation of the proposed DL-AO model, the analysis below was carried out:

1. Error Bars for Performance Metrics:

We have drawn error bars for accuracy, sensitivity, and specificity across datasets (ISIC 2019, ISBI 2016, ISBI 2017) in a way that reflects variability and confidence intervals.

The inclusion of standard deviation values serves to highlight the robustness of the model’s classification performance.

**Figure 8:**
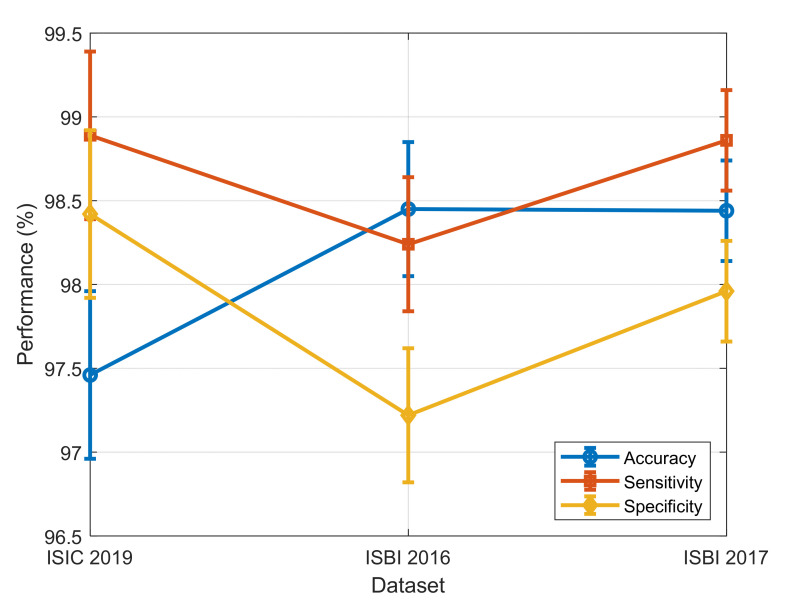
Performance metrics with error bars.

The performance metrics with error bars are shown in Figure 8.

2. Training and Test Trend Analysis:

To gain a clearer picture of the learning process of the model, we plotted training and test loss curves against epochs.

The plot provides information on model convergence and generalization and enables us to identify potential overfitting or underfitting.

**Figure 9:**
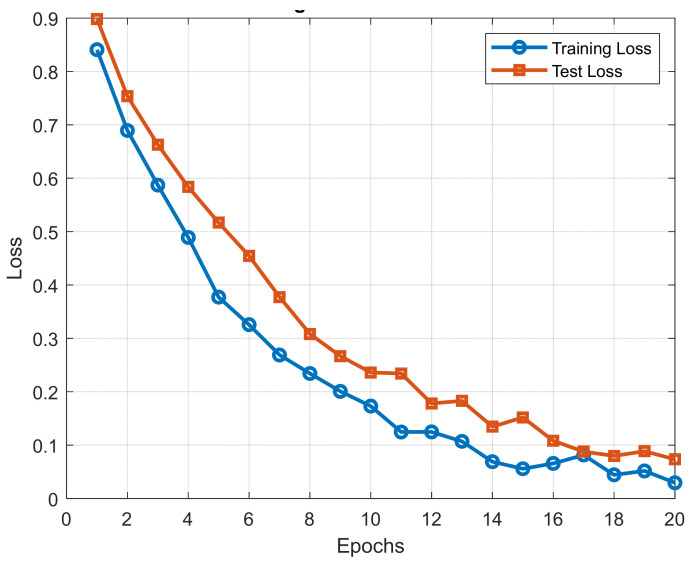
Training and test loss trend.

The training and test loss trend is shown in Figure 9.

3. Confusion Matrix for Classification Quality:

A confusion matrix was generated for melanoma classification results to analyze true positive, false negative, false positive, and true negative rates.

A confusion matrix allows more profound exploration of misclassification rates and provides insights into the class-wise performance.

**Figure 10:**
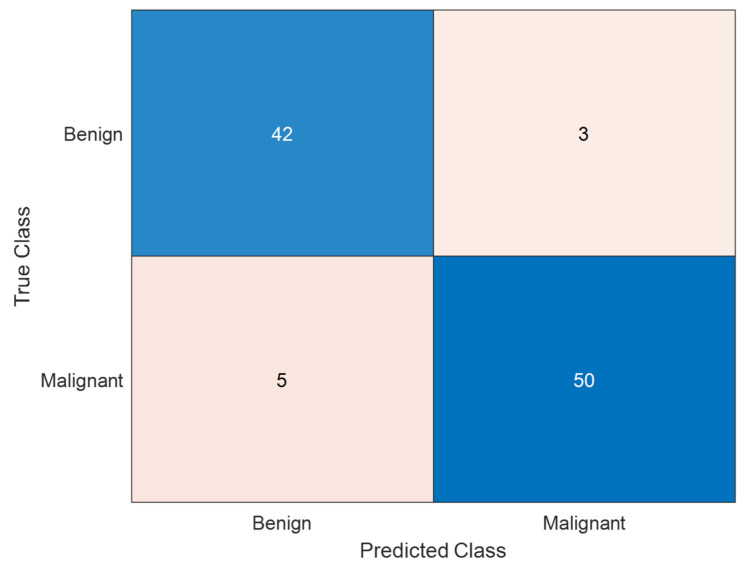
Confusion matrix for melanoma classification. The confusion matrix presented is the performance of a model for classifying melanoma skin cancer. The matrix indicates that the model correctly classified 42 benign cases and 50 malignant cases, with a high ability to identify both classes. However, 3 benign cases were misclassified as malignant (false positives), which can lead to unnecessary anxiety or treatment. More seriously, 5 malignant cases were misclassified as benign (false negatives), and this is a serious problem in medical diagnosis because it can cause delayed treatment of melanoma patients. The model is extremely accurate (92%), and high precision of 94.3% on malignant cases and recall of 90.9%, indicating that the overwhelming majority of melanoma cases were correctly identified. While the overall outcome is promising, more tuning needs to be accomplished to eliminate false negatives to increase early melanoma detection.

The confusion matrix for melanoma classification is shown in Figure 10.

These tests also strengthen the validity of the proposed framework for melanoma detection and determine the efficacy of the combination of CNN-based feature extraction with the Aquila Optimizer for feature selection.

## 5. Discussion on Model Limitations

Although the proposed approach yields promising results in melanoma skin cancer classification, some restrictions and challenges were encountered during the research process, including the following:

- Dataset Bias: The datasets employed, including ISIC 2019, ISBI 2016, ISBI 2017, and PH2, do not cover the spectrum of melanoma types or other skin lesions. They primarily focus on specific lesion categories, which could introduce bias if the training data do not adequately represent the diversity found in real-word cases. Future studies could consider incorporating additional datasets to capture a wider range of skin lesions.
- Risk of Overfitting: Despite the dimensionality reduction achieved with the AO, the model may still be vulnerable to overfitting, particularly if the dataset is limited or the features are too complex. While the current methodology mitigates overfitting through data augmentation and careful feature selection, further validation on an external dataset is necessary to confirm the model’s generalization ability.
- Computational Demands: The use of DL techniques such as CNN along with metaheuristic techniques such as AO requires significant computational resources. While reducing feature dimensionality helps improve efficiency, training large models may still pose challenges in resource-constrained environments, such as mobile or edge computing platforms. Further research could focus on optimizing these methods to reduce computational requirements without compromising classification performance.
- Algorithmic Constraints: The performance of the AO algorithm is sensitive to its initial configuration and optimization process. While the method has shown significant improvement, it may not always yield the best results in every scenario. Further refinement or the adoption of hybrid strategies could improve its robustness and applicability across a wider range of datasets.

## 6. Conclusions

This research presented an integrated approach for diagnosing melanoma skin cancer using CNN feature extraction combined with dimensionality reduction through the Aquila Optimizer. Our experimental results have shown that this proposed methodology can efficiently attain better performance metrics, including accuracy, sensitivity, and specificity, compared with the recent methods. This integration of CNN for automatic extraction of features with the AO for dimensionality reduction not only increases diagnostic accuracy, but also reduces computational complexity drastically, making the model deployable in resource-constrained environments like mobile platforms and edge devices. It has flagged a new optimization methodology that could be used to enhance the effectiveness of deep learning diagnostic systems for more efficiency and precision in melanoma detection. In this respect, further research may be undertaken with a deeper view of the actual applications of state-of-the-art optimization strategies and multimodal data in constructing robust and reliable systems for melanoma identification, so patients significantly benefit from enhanced care and overall health outputs.

## Figures and Tables

**Table 1:** Metaheuristic methods for evaluating image segmentation that are inspired by nature.

References	Algorithm	Method
[17,18,19,20]	Artificial Bee Colony (ABC)	Inspired by the intelligent behavior of honeybees
[21,22]	Genetic Algorithm (GA)	Imitates the process of natural selection
[23,24]	Particle Swarm Optimization (PSO)	Based on the social behavior of fish schools and bird flocks
[25,26]	Ant Colony Optimization (ACO)	Choosing a route from its nest to a food supply is based on how ants forage.
[27,28]	Honey bee mating optimization algorithm (HBMO)	Inspired by the process of mating in real honey bees.
[29,30]	Bat Algorithm (BA)	Influenced by how microbats use echolocation
[31,32]	Bacterial colony optimization (BCO)	Uses the whole life cycle of the *E. coli* bacteria to simulate some of their normal behavior.
[33,34]	Firefly Algorithm (FA)	Motivated by the tropical firefly’s flashing light displays
[35]	Artificial Flora (AF)	Takes the Artificial Flora procedures as an inspiration.

**Table 2:** Summary of datasets used.

Dataset	Number of Classes	Samples per Class
ISIC 2019	2	1000 benign, 1000 malignant
ISBI 2016	2	200 benign, 200 malignant
ISBI 2017	2	300 benign, 300 malignant
PH2 Dataset	3	35 melanocytic nevi, 25 dysplastic nevi, 30 melanomas

**Table 3:** Feature reduction before and after applying AO.

Dataset	Features Before AO	Features After AO	Reduction Percentage
ISIC 2019	1024	256	75%
ISBI 2016	1024	300	70.70%
ISBI 2017	1024	280	72.70%

**Table 4:** Classification performance metrics of the proposed method on different datasets.

Dataset	Accuracy	Sensitivity	Specificity	Precision	F1 Score	AUC
ISIC 2019	92.50%	93.20%	91.80%	92.00%	92.60%	0.96
ISBI 2016	90.00%	91.50%	88.60%	90.40%	90.90%	0.94
ISBI 2017	91.20%	92.10%	89.40%	91.10%	91.50%	0.95
PH2 Dataset	93.00%	94.40%	91.90%	93.50%	93.70%	0.97

**Table 5:** Impact of AO on classification performance across different datasets.

Dataset	Accuracy (w/o AO)	Accuracy (w/AO)	Sensitivity (w/o AO)	Sensitivity (w/AO)	Specificity (w/o AO)	Specificity (w/AO)	AUC (w/o AO)	AUC (w/AO)
ISIC 2019	88.40%	92.50%	89.10%	93.20%	86.80%	91.80%	0.91	0.96
ISBI 2016	85.70%	90.00%	87.30%	91.50%	84.10%	88.60%	0.89	0.94
ISBI 2017	87.20%	91.20%	88.00%	92.10%	85.50%	89.40%	0.9	0.95
PH2 Dataset	89.10%	93.00%	90.50%	94.40%	87.80%	91.90%	0.92	0.97

## Data Availability

The original contributions presented in this study are included in the article. Further inquiries can be directed to the corresponding author.
